# Overcooling of offices reveals gender inequity in thermal comfort

**DOI:** 10.1038/s41598-021-03121-1

**Published:** 2021-12-08

**Authors:** Thomas Parkinson, Stefano Schiavon, Richard de Dear, Gail Brager

**Affiliations:** 1 Center for the Built Environment (CBE), University of California Berkeley, Berkeley, CA USA; 2 Indoor Environmental Quality Lab, School of Architecture, Design and Planning, The University of Sydney, Sydney, NSW 2006 Australia

**Keywords:** Energy and society, Psychology and behaviour, Sustainability

## Abstract

Growth in energy use for indoor cooling tripled between 1990 and 2016 to outpace any other end use in buildings. Part of this energy demand is wasted on excessive cooling of offices, a practice known as overcooling. Overcooling has been attributed to poorly designed or managed air-conditioning systems with thermostats that are often set below recommended comfort temperatures. Prior research has reported lower thermal comfort for women in office buildings, but there is insufficient evidence to explain the reasons for this disparity. We use two large and independent datasets from US buildings to show that office temperatures are less comfortable for women largely due to overcooling. Survey responses show that uncomfortable temperatures are more likely to be cold than hot regardless of season. Crowdsourced data suggests that overcooling is a common problem in warm weather in offices across the US. The associated impacts of this pervasive overcooling on well-being and performance are borne predominantly by women. The problem is likely to increase in the future due to growing demand for cooling in increasingly extreme climates. There is a need to rethink the approach to air-conditioning office buildings in light of this gender inequity caused by overcooling.

## Introduction

The well-being and performance of office workers relies in part on satisfactory indoor environments. Delivering comfortable and healthy offices is too often done in an energy-intensive manner, leading to increasing cooling demand globally^1^. One way to reduce building energy consumption is to minimize heating, ventilation, and air-conditioning (HVAC) usage, which represents the largest source of energy use in buildings^2^. Reducing HVAC energy without compromising comfort is difficult to accomplish because occupants need a range of different temperatures to be comfortable^3^ while buildings are typically designed and operated to provide homogeneous conditions. Dissatisfaction with temperature ranks as the second most frequent challenge with office environments behind acoustics^4^, and much of this dissatisfaction is attributable to excessive cooling. This issue is known as overcooling and is estimated to cost $10B in wasted electricity annually^5^. Overcooling poses a societal-scale barrier to improving the well-being and performance of the US workforce, and curbing greenhouse gas emissions and energy profligacy in the built environment.

Ranges of comfortable indoor temperatures are shaped by prevailing weather and differ across seasons and climates^6^. This means that comfort temperatures generally increase in summer and decrease in winter. For example, the dominant standard for thermal comfort^7^ suggests a summer comfort temperature range that is approximately 1.5 °C warmer than the range in winter. Yet, paradoxically, indoor temperatures in US offices have been shown to be lower in summer than winter^8^. Similar overcooling practices have been reported in tropical climates throughout Asia^9,10^ and the Middle East^11^, and are forecast to increase in the future^1^. The cause of overcooling is often attributed to two broad issues. First, office buildings with suboptimal HVAC design and control strategies for their local climates^12^. Second, the most popular model of human comfort for designing HVAC systems—the predicted mean vote or PMV model—tends to overestimate discomfort in warm temperatures^13^. Advocates for cooler-than-comfortable offices point to expected performance improvements, but the empirical evidence supporting that relationship is questionable^14^.

Most research evidence on gender differences in thermal comfort would suggest that overcooling is likely to affect women more than men. Laboratory studies of temperature preferences by gender report the largest differences in cold environments^15^. Reasons for higher dissatisfaction in cold temperatures for women have been attributed to physiological and clothing differences. A biophysical analysis showed an underestimation of the metabolic rate of women in the heat-balance model codified by international thermal comfort standards^16^. The outcome of a systematic error like this would be the specification of cooler temperatures that align with the typical metabolic heat produced by men. Field studies confirm this pattern, with women reporting lower thermal satisfaction overall^17^ but particularly in summer^18^. This gender gap in comfort widens when larger groups of people share one thermostat controlling the same space^19^. Greater dissatisfaction for women occurs despite them being more compromising than men when resolving conflicts^20^. This leads to less favorable outcomes in comfort negotiations^21^.

Extant evidence demonstrates that temperatures in many US office buildings do not reflect the thermal preferences and requirements of women. Yet the reasons behind this gender disparity have not been addressed directly. The typical explanations found in thermal comfort literature—lower metabolic rate or lighter clothing—inadvertently position women as the source of the problem rather than the thermal environments of offices. We argue that current air-conditioning strategies tend to overcool office buildings, leading to the lower thermal satisfaction for women reported in the literature. This disparity is likely to be most evident during summertime overcooling events when seasonal expectations are shifted in the warmer direction, occupants are more lightly dressed, but indoor temperatures are lower. We use two large datasets to highlight the gender inequity of overcooling in US offices as well as its prevalence. Identifying the reasons for differences in comfort outcomes for women and men is needed to redefine standard indoor temperatures to reduce overall thermal dissatisfaction in offices.

**Figure 1:**
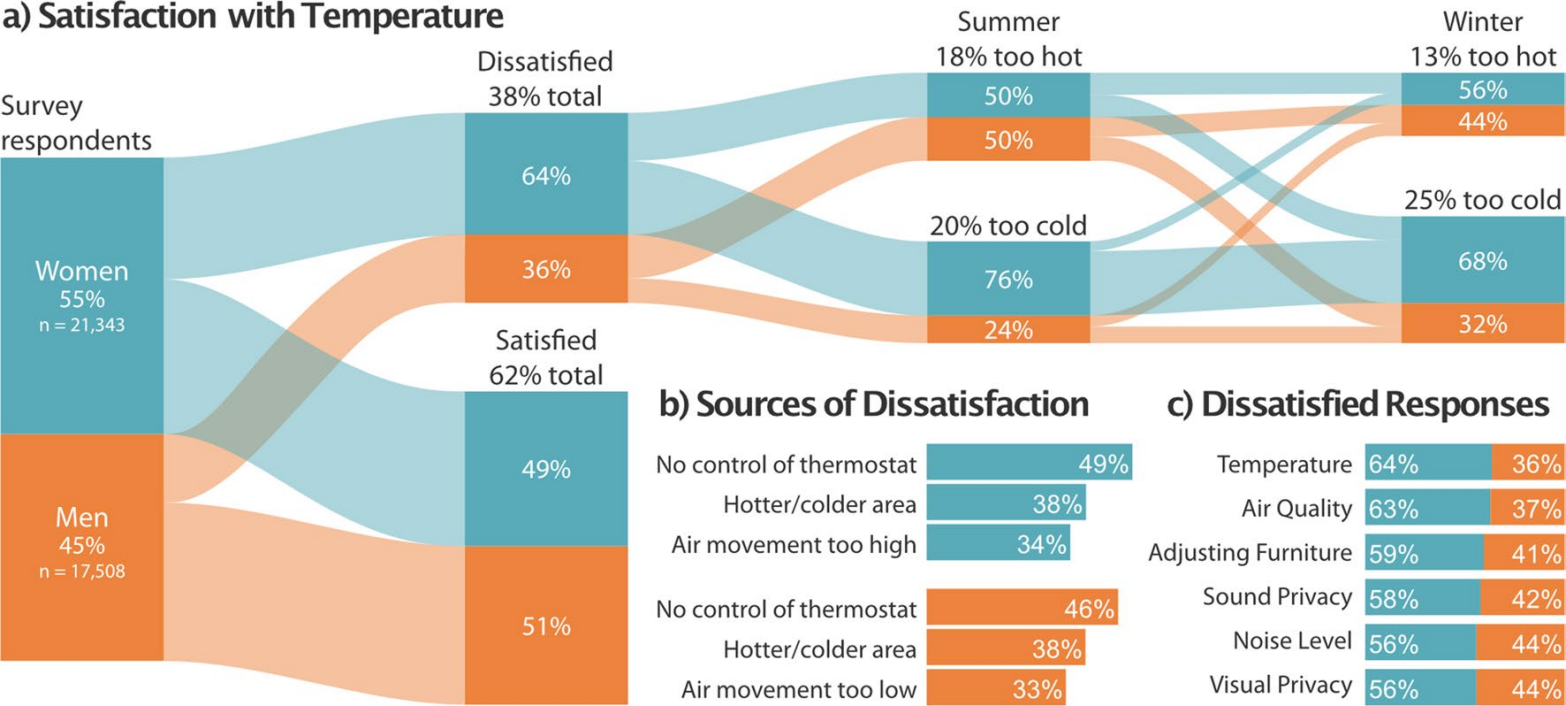
Survey responses about office temperatures. Summary of 38,851 responses to the CBE Occupant Survey. (**a**) Breakdown of satisfaction with temperature in office buildings by gender. Percentage of respondents are given for each question. Office temperatures were more satisfactory for men than women, who comprise the majority of occupants reporting feeling too cold in winter and summer. The alluvial plot was produced using^29^. (**b**) Three most frequent reasons for the source of dissatisfaction with temperature for men and women as the percentages of dissatisfied respondents. The first two are the same between genders, but the third—air movement too high/too low—is in opposition. (**c**) Dissatisfaction with the six lowest-scoring workspace items from the CBE Occupant Survey. Temperature and air quality had the largest divide between women and men; most other items resembled the gender proportions of survey respondents.

We analyzed 38,851 responses to questionnaire items in the CBE Occupant Survey^4^ about satisfaction with temperature from 435 office buildings across 168 cities in the US. The results in Fig. 1 show 38% of respondents were dissatisfied with the temperature in their office, far worse than the 20% dissatisfaction limit set in thermal comfort standards^7^. When these data were broken down by gender, women accounted for nearly two-thirds of those dissatisfied respondents. The results of a logistic regression indicate that office temperatures were 1.8 times more likely to be dissatisfactory for women (*b* = 0.58, 95% CI [0.54, 0.61], *p* ≤ 0.001) than men. The gender proportions of dissatisfied respondents were markedly different for temperature and air quality compared to the surveyed items exploring other indoor environmental quality parameters in Fig. 1c. Similar proportions for air quality may be due to the impact of temperature on perceived air quality^22^. Respondents were also asked to rate whether their thermal comfort enhanced or interfered with their ability to get their job done (not shown in figure). There was a high positive correlation (r = 0.85, *p* ≤ 0.01) between satisfaction with temperature and respondents’ belief that thermal comfort influenced their ability to get their work done. More men (44%) reported temperature enhancing their self-reported performance than women (31%). In fact, the thermal environment interfered with the performance of 42% of women; 27% of both men and women reported no effect.

Those expressing dissatisfaction were asked to evaluate their office temperature in summer and winter. The temperature in winter was more often ‘too cold’ than ‘too hot’ for those occupants, as expected on the basis of outdoor conditions. Respondents were more likely to report temperatures in summer being too cold, indicative of the issue of overcooling and a disconnect with the outdoor environment. Most dissatisfied occupants had the same response for both seasons, implying sustained cold discomfort year-round. Persistently cold offices disproportionately affected women, particularly in summer when only 24% of complaints of cold temperatures came from men. Women who were dissatisfied with the temperature in their office were over three times more likely to feel too cold in summer compared to their male counterparts. The most common source of dissatisfaction for both men and women was a perceived lack of control over the temperature. One third of men thought the rate of air movement was too low while one third of women thought it was too high. This reflects the different thermal experiences and preferences of men and women in offices. Inflexible clothing policy was the least common reason offered by either gender.

**Figure 2:**
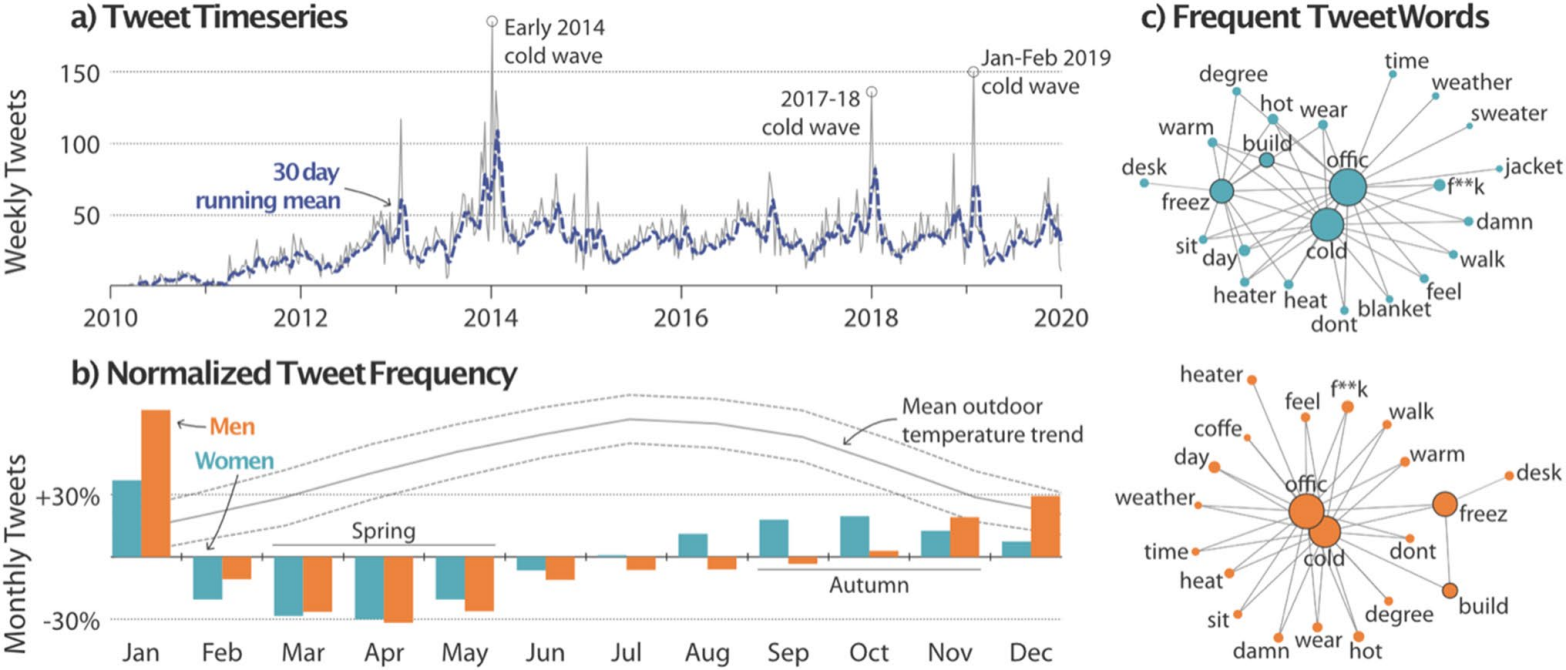
Cold office tweets. Twitter activity from US office occupants who are cold in their workspace. (**a**) The weekly number of tweets over the 10-year period that met the criteria for inclusion in this study. Notable meteorological events are labelled and show the corresponding increase in cold office tweets. (**b**) Normalized monthly tweet activity by gender. We calculated the change in Twitter activity each month relative to the total count of cold office tweets divided by the number of months. There is a relative increase in activity starting in July for women and in October for men. Both genders have the highest number of cold office tweets in January. The mean (solid), minimum (dashed), and maximum (dashed) outdoor temperature trend is shown for reference. (**c**) Network graph of the paired word frequencies of the cold office tweets for women (top) and men (bottom). Search keywords (office, cold, building, freezing) are outlined in grey and sit at the center of the network plot. More frequent words have larger points and less frequent words have smaller points; search keywords were most frequent. Lines show the common term pairs between frequently used words. The layout of points is to reduce overlap and is not statistically meaningful. Many term pairs are similar by gender, but women discussed cold weather clothing items like sweater, jacket, and blanket whereas men did not. Network graph produced using^38^.

The CBE Occupant Survey database is subject to self-selection bias and may not be representative of US offices. To complement and expand the analysis beyond our survey database, we used crowd-sourced thermal comfort feedback from office building occupants on Twitter. We collected 16,791 tweets with common expressions of cold discomfort in US offices between January 2010 and December 2019. The highest number of weekly tweets shown in Fig. 2a occurred during extreme cold weather events predominantly in the Northeast and Midwest regions. While a similar number of tweets were sent in Spring and Winter months, tweets in winter were overwhelmingly sent in January during extreme weather events. This coincides with the return to offices after the holiday shutdown when heating systems are throttled, internal heat gains are largely removed, and building thermal mass is chilled. The number of cold office tweets increase again at the onset of summer from their lowest levels in Spring. Temporal trends demonstrate the relationship between crowdsourced data (tweets) and weather that can be used to study temperature-related phenomenon.

Overall, there were more tweets about cold offices from women (66%) than men (34%). This is an overrepresentation given that an estimated 55% of US Twitter users are women^23^. Moreover, the seasonal trend in tweet activity differs between men and women. The sinusoidal trend in monthly tweets in Fig. 2b from women increases in summer and demonstrates an out-of-phase seasonality with outdoor temperature. In contrast, tweets about cold offices from men exhibit less pronounced seasonality and only increase in winter. The decreased twitter activity from men in summer suggest the cold indoor temperatures are more favorable for men. This may be partially attributable to greater adaptability to the outdoor conditions in the clothing worn by women and or their temperature expectations. Clothing items do appear frequently in cold office tweets from women but are not common in tweets from men (Fig. 2c). Evidence of meaningful differences in the summer office attires of men and women is lacking^24^. Nevertheless, clothing differences are not a sufficient reason for office temperatures to disproportionately impact a particular group of occupants.

**Figure 3:**
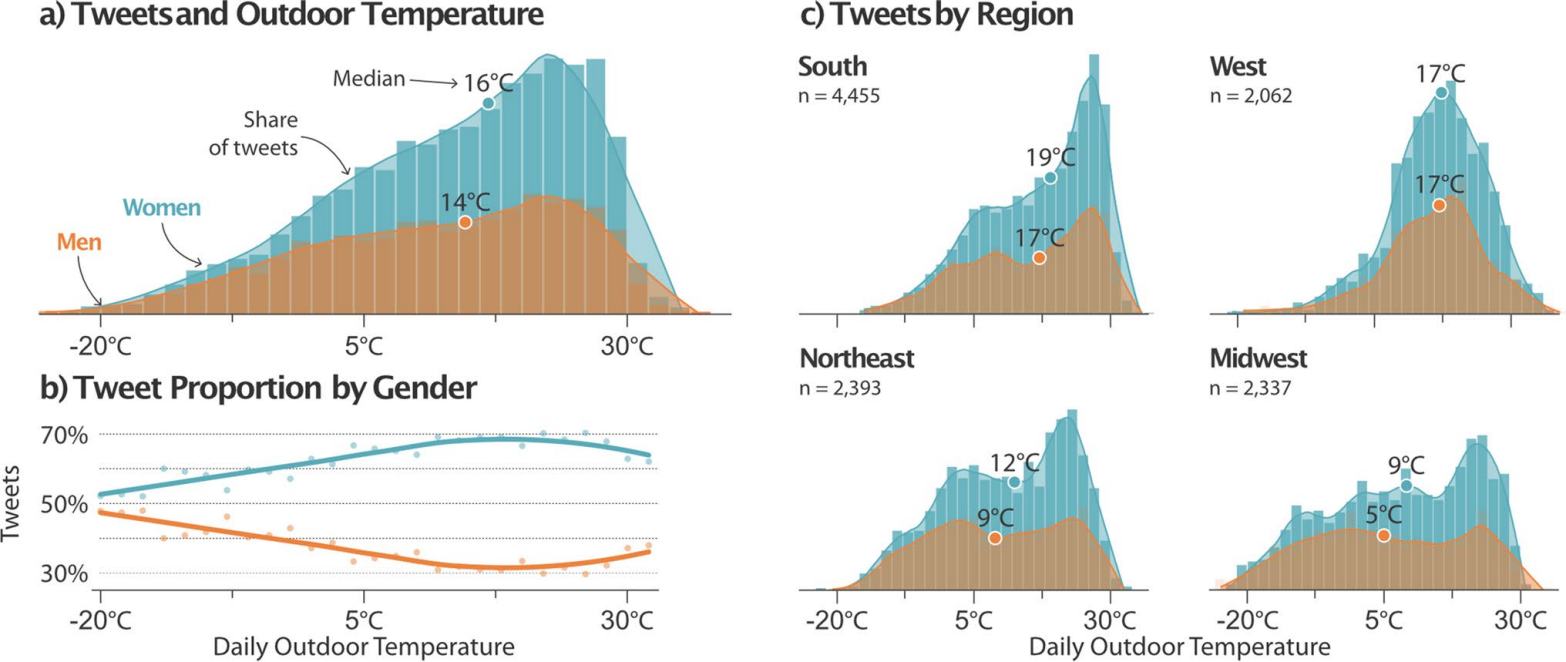
Cold office tweets and outdoor temperature. The distribution of cold office tweets and average daily outdoor temperature (2 °C bin width) where the tweet was sent. (**a**) Distribution of tweets for women and men across the range of outdoor temperatures. There are more tweets from women at all temperatures. The median outdoor temperature is shown inset and is lower for men than women, indicating that a greater proportion of tweets from women are in warmer outdoor temperatures than men. (**b**) The proportion of tweets from balanced samples (repeated 100 times) of binned outdoor temperatures. The likelihood of women tweeting about cold discomfort increases with warmer temperatures. (**c**) Tweet distribution of men and women grouped by US Census region. Southern states had the most tweets, which likely reflects the excessive cooling of offices in part from the dehumidification requirements of those climates.

We grouped tweets based on location and binned them on the average outdoor temperature on the day to explore differences in the relationship between weather and cold complaints. Figure 3a shows that cold office tweets were common when daily temperatures were above 20 °C, and the majority were from women. The median outdoor temperature that splits the distribution in half is 2.2 °C higher for women than men (Mood’s median test, *p* ≤ 0.001). Furthermore, the likelihood of cold office tweets originating from women increases in warmer temperatures (Fig. 3b). Different distributions between regions shown in Fig. 3c reveals the mediating effects of climate. Occupants in the South contributed the largest portion of tweets about overcooling in warm temperatures. This likely reflects overcooling from the dehumidification requirements of those local climates. Regions with distinct seasons—Midwest and Northeast—had frequent cold complaints in both cold and warm temperatures. The moderate climates of large coastal cities in the West coast likely explain the normal distribution. These results show that although the phenomenon of overcooling varies slightly with climate, the unseasonably cold indoor temperatures consistently affect the thermal satisfaction of women.

Our analysis of the CBE Occupant Survey presented evidence that cold indoor temperatures in office buildings favor the preferences of men and lead to relatively higher levels of dissatisfaction for women. Crowdsourced data from Twitter confirmed this conclusion and provided a novel approach to expanding the generalizability of findings from post-occupancy evaluations. We showed that overcooling is a widespread problem that hinders the push for more balanced and equitable workplaces. These findings corroborate existing evidence of lower thermal satisfaction in offices for women and identify overcooling as a common reason for the gender disparity. In addition to a wasteful and unsustainable energy expense^25^, this thermal inequity may adversely impact the ability of women to focus on their work.

It is not possible to offer precise reasons for overcooling from our dataset. However, prior research provides useful insight into the potential causes. Two common issues in the operation of air conditioning systems in commercial buildings are likely to contribute to inequitable office temperatures: (1) lower-than-necessary setpoint temperatures that favor the thermal preferences of men, and (2) misconfigurations in HVAC systems, particularly in warm and humid climates. For the first issue, simply increasing office thermostats in summer to align with thermal comfort guidelines^7^ would help address any gender bias in setpoint temperatures. Potential increases in thermal discomfort due to higher temperature can be readily compensated with air movement^26^. For the second issue, technological fixes to existing building control systems that target minimum airflow rates and supply air temperature controls can improve thermal satisfaction in summer while reducing HVAC energy use^12^. Finally, personal comfort systems are a technological solution to delivering conditions to suit individual thermal preferences without making the office uncomfortable for others^27^. These solutions would help mitigate the reliance of US offices on cooling and ensure comfortable office environments that support more occupants in their work.

## Methods

Field studies of thermal comfort are normally conducted in offices using in situ temperature measurements paired with surveys to determine occupant thermal comfort. This is a reliable method but is logistically constrained and difficult to scale. We combined a traditional post occupancy evaluation (CBE Occupant Survey) with a novel crowdsourcing approach to collecting occupant comfort assessments (Twitter) in office buildings. All analyses were done in R (version 4.2) and RStudio (version 1.4.1103) along with ‘tidyverse’^28^ suite of packages for data wrangling and plotting; additional packages were used as described in the following sections.

### CBE occupant survey

The CBE Occupant Survey is a web-based post occupancy evaluation tool designed to assess satisfaction with workplace design. It has almost 90,000 responses from approximately 900 buildings; the database was recently summarized by^4^. Respondents are asked a series of questions related to the indoor environment of their workplace. We focused on responses to the question asking occupants to evaluate their satisfaction with the temperature in their office (7-point Likert-type satisfaction scale). If dissatisfied, they are asked to indicate the reason for their dissatisfaction from a checklist and nominate whether they were too cold or too hot in summer and winter. The gender of respondents is sometimes excluded from surveys due to the potentially sensitive nature of the question. There was a total of 38,851 responses from surveys between March 2000 and December 2019 after filtering for the requisite data. Most surveys in the dataset were from buildings in the Western states (e.g., 45% California, 6% Washington, 6% Oregon), with fewer in the Midwest (16%), South (14%) and Northeast (6%). We compared the thermal satisfaction of occupants between regions as well as age groups but did not find meaningful differences. We used the ‘ggalluvial’ package^29^ to generate the alluvial plot, and the ‘base R’ implementation of binomial logistic regression to model the probabilities of a given outcome.

### Twitter

Twitter is a text-based social network where users ‘tweet’ their thoughts and feelings. It is used frequently in research in other disciplines like political and computer science, and has been used for other temperature-related studies^30,31^. We used the ‘rtweet’ package^32^ for retrieving results from their full archive API endpoint using the academic research product. We used keyword searches containing the words “freezing OR cold” AND “office OR desk OR building” to return tweets from January 1st, 2010 to December 31st, 2019. Negative keywords like “apartment”, “house” and “government” helped to filter out irrelevant tweets. “Cold” was chosen as a keyword instead of “cool” as it is largely understood as describing temperatures outside the comfort range^33^. Retweets were ignored along with tweets linking to news articles. Results were limited to geolocated tweets from the United States. This substantially reduced the sample size as it requires users to actively share their location. However, it was required for our bounded analysis of US office buildings as well as pairing with meteorological data.

The resulting database contained 16,791 tweets that met the criteria for inclusion in our study. We added contemporaneous outdoor temperature from the nearest weather station using the ‘rnoaa’ package^34^. Daily climate summaries were retrieved from the National Oceanic and Atmospheric Administration’s (NOAA) Global Historical Climatology Network-Daily (GHCN-D) database. Tweet coordinates and timestamp were used to find the nearest meteorological station and retrieve the archived minimum and maximum daily outdoor temperatures on the day of the tweet (see Fig. S1). We calculated the average daily temperature as the mean of daily minimum and maximum temperatures. Over 75% of the meteorological stations were less than 10 km away from the location of the Twitter user on the day of the tweet (see Fig. S2).

Twitter does not publish the gender of users. We used the ‘gender’ package^35^ to infer the likely gender of a Twitter user based on their username. The first word with three or more characters was extracted from their username (after removing stop words) and input into the gender prediction algorithm using the ‘ssa’ method to infer the likely gender. This method is based on the Social Security Administration (SSA) Baby Names list. We assumed users were less than 65 years old and set the minimum and maximum year as 1945 and 2005 respectively. This step reduced the size of our database to 11,247 tweets due to unidentifiable names or weak gender predictions.

We randomly sampled 100 tweets from our database to manually verify our methods. Of the sample, 85 were directly discussing a cold office and 6 were discussing cold weather and the office. 2 were referring to having a cold either from or in the office. The remaining 7 tweets were entirely unrelated to a cold office. Manual inspection of gender based on the 100 sampled user profiles found 93 gender predictions were likely to be correct. 6 of the 7 incorrect predictions were women being classified as men.

We used the tweet timestamp and coordinates to determine the local time of the tweet. Using this data, we found that 94% of tweets were made on weekdays and 87% between 8 A.M. and 6 P.M. (see Fig. S3). This suggests most tweets were made during normal business hours. For the text mining of tweets, we used the ‘tidytext’ package^36^ for tokenizing words and the ‘SnowballC’ package^37^ for word stemming. Stop words were removed before counting word pairs based on the stemmed words. The network graph (Fig. 2c) was produced using the ‘ggraph’ package^38^ based on the top 10% of frequent word pairs.

### Limitations

There are many limitations when using crowd-sourced data and inferring sociodemographic parameters. Specifically, we did not verify that all the tweets were related to cold offices. We were limited to manual inspection of a sample of tweets. We consider 85% to be an acceptable true positive rate given the geolocation requirement likely under-sampled the relevant tweets in the full Twitter archive. We acknowledge that the binary treatment of gender is a simplification that ignores the fact that gender is not dichotomous. Furthermore, estimating gender based on a name is prone to error and name may not represent gender identity. However, manual verification of labeled gender found an accuracy of 93% which we deemed to be sufficient for our study. Investigation of the false classifications suggest that errors in the gender prediction are likely to have underestimate the proportion of women in our dataset. Lastly, the language and topics discussed on Twitter can vary by gender^39^. It is possible that these lexical variations exist within our database of tweets that better explain the gender proportions. This is an important consideration but outside the project scope and we therefore did not test for it.

### Ethics statement

The CBE Occupant Survey was approved by the IRB at the University of California, Berkeley (grant number IRB-2010-05-1550). Responses were anonymous and informed consent was obtained from survey participants. All research was conducted in accordance with the principles of the Belmont Report.

## Supplementary Information

Supplementary Figures.

## Data Availability

Data were not deposited to any database repositories. The CBE Occupant Survey database is not available for open access to comply with the approved IRB protocol at the University of California, Berkeley (Grant number IRB-2010-05-1550). In compliance with Twitter’s Terms of Service, the authors cannot share the database of tweets. Data summaries related to this paper may be requested from the authors.
