# Supplementary material for: Overcooling of offices reveals gender inequity in thermal comfort
Source: Sci Rep. 2021 Dec 8;11:23684. doi: 10.1038/s41598-021-03121-1 (PMC8655064; doi:10.1038/s41598-021-03121-1)
Supplement: Supplementary file 1 — Supplementary Figures. [file 41598_2021_3121_MOESM1_ESM.pdf]

Supplementary Material: Overcooling of offices reveals gender inequity in thermal comfort

Thomas Parkinson, Stefano Schiavon, Richard de Dear, Gail Brager

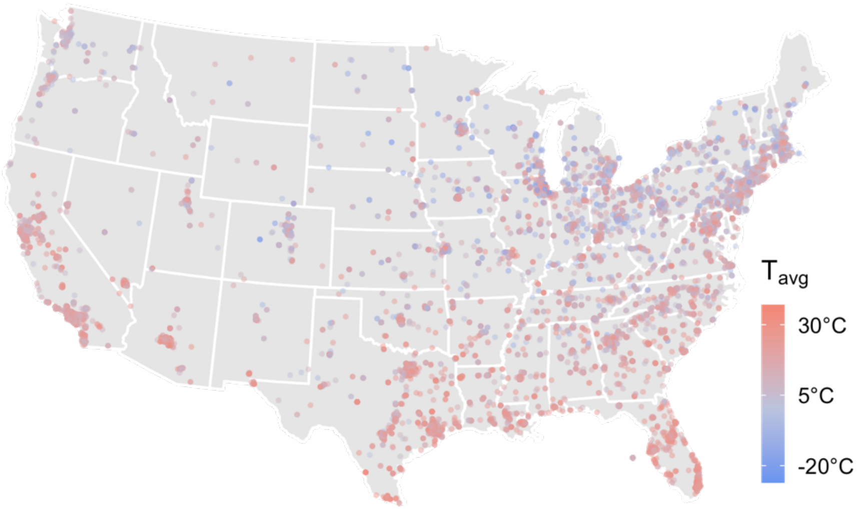

Figure s1. Location and daily average outdoor temperature for the cold office tweets in our dataset. The map was produced using the 'maps' package (version 3.3.0, https://CRAN.R-project.org/package=maps) in R.

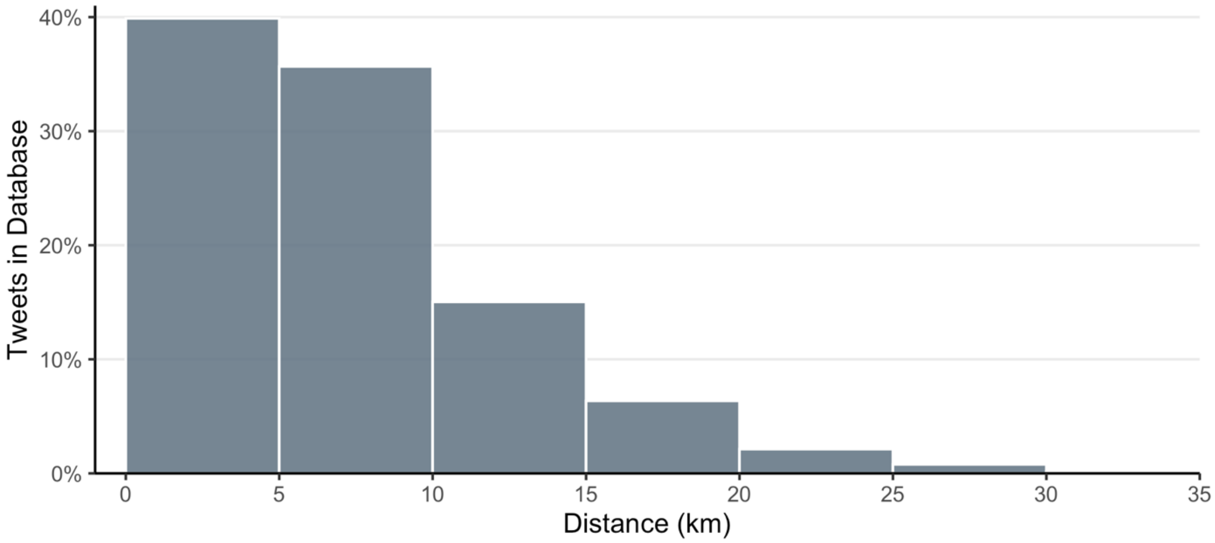

Figure s2. The distribution of distances between the precise tweet location and the closest meteorological stations used to source outdoor temperature data. 76% of tweets with associated meteorological data were paired with stations less than 10 km away.

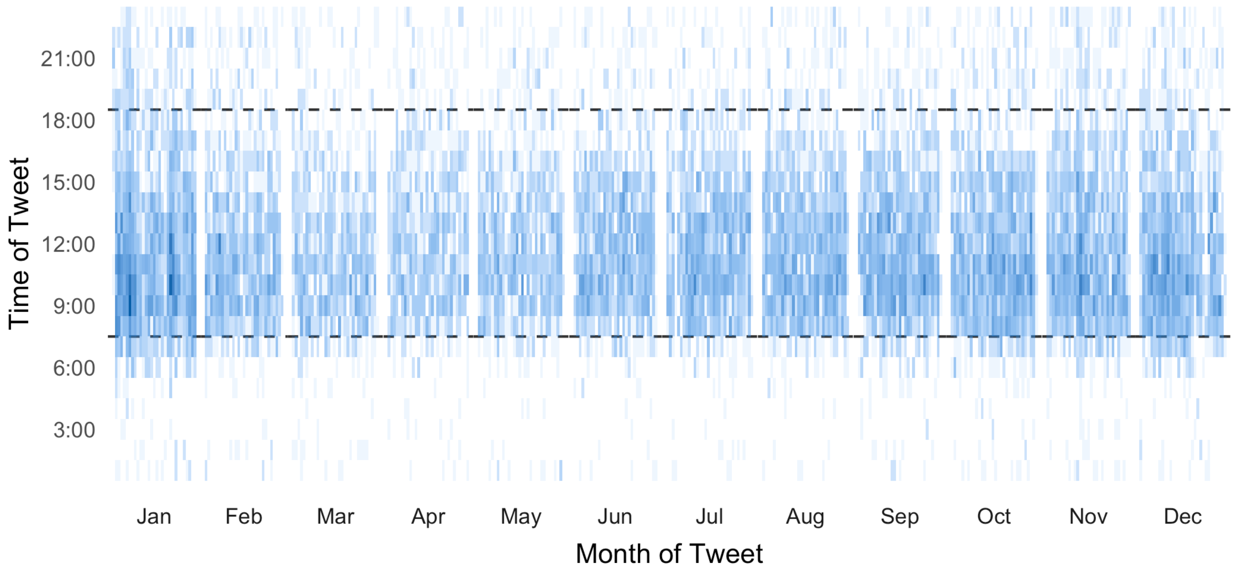

Figure s3. The time and month of the cold office tweets used in our analysis. The saturation of the tiles is set by the number of tweets on that day of the year. Regular office hours (8 A.M. to 6 P.M.) are shown by the dashed lines.
